# Estimating the impact of implementing an integrated care management approach with Atrial fibrillation Better Care (ABC) pathway for patients with atrial fibrillation in England from 2020 to 2040

**DOI:** 10.1093/ehjqcco/qcad055

**Published:** 2023-09-11

**Authors:** Elizabeth M Camacho, Gregory Y H Lip

**Affiliations:** Institute of Population Health, University of Liverpool, UK; Liverpool Centre for Cardiovascular Science at University of Liverpool, Liverpool John Moores University and Liverpool Heart & Chest Hospital, Liverpool, UK; Liverpool Centre for Cardiovascular Science at University of Liverpool, Liverpool John Moores University and Liverpool Heart & Chest Hospital, Liverpool, UK; Danish Center for Health Services Research, Department of Clinical Medicine, Aalborg University, Aalborg, Denmark

**Keywords:** Atrial fibrillation, ABC pathway, Costs

## Abstract

**Background:**

Stroke prevention is central to the management of atrial fibrillation (AF), but there remains a residual risk of adverse outcomes in anticoagulated AF patients. Hence, current guidelines have proposed a more holistic or integrated approach to AF management, based on the Atrial fibrillation Better Care (ABC) pathway, as follows: (A) avoid stroke with anticoagulation; (B) better symptom control with patient-centred symptom directed decisions on rate or rhythm control; and (C) cardiovascular and comorbidity management, including lifestyle factors. There has been no formal healthcare cost analysis from the UK National Health Service (NHS) perspective of ABC pathway implementation to optimize the management of AF. Our aim was to estimate the number of patients with AF in the UK each year up to 2040, their morbidity and mortality, and the associated healthcare costs, and secondly, to estimate improvements in morbidity and mortality of implementing an ABC pathway, and the impact on costs.

**Results:**

In 2020, there were an estimated 1 463 538 AF patients, resulting in £286 million of stroke care and £191 million of care related to bleeds annually. By 2030, it is expected that there will be 2 115 332 AF patients, resulting in £666 million of stroke healthcare and £444 million of healthcare related to bleeds. By 2040, this is expected to rise to 2 856 489 AF patients, with £1096 million of stroke healthcare and £731 million of healthcare related to bleeds for that year. If in 2040 patients are managed on an ABC pathway, this could prevent between 3724 and 18 622 strokes and between 5378 and 26 890 bleeds, and save between 16 131 and 80 653 lives depending on the proportion of patients managed on the pathway. This would equate to cost reductions of between £143.9 million and £719.6 million for the year.

**Conclusion:**

We estimate that there will be a substantial healthcare burden in the UK NHS associated with AF, from strokes, bleeds, and mortality over the next decades. If patients are managed with a holistic or integrated care approach based on the ABC pathway, this could prevent strokes and bleeds that equate to substantial NHS healthcare cost reductions, and save lives.

KEY LEARNING POINTS What is already known:

- Current guidelines have proposed an integrated approach to atrial fibrillation (AF) management, based on the Atrial fibrillation Better Care (ABC) pathway, as follows: (A) avoid stroke with anticoagulation; (B) better symptom control with patient-centred symptom directed decisions on rate or rhythm control; and (C) cardiovascular and comorbidity management, including lifestyle factors.
- There has been no formal healthcare cost analysis from the UK National Health Service (NHS) perspective of ABC pathway implementation to optimize the management of AF.

What this study adds:

- We estimate a substantial healthcare burden of AF patients, from strokes, bleeds, and mortality in the UK NHS over the next decades.
- If patients are managed based on the ABC pathway, this management approach could prevent strokes and bleeds, and save lives that equate to substantial NHS healthcare cost reductions.

## Introduction

As the commonest sustained cardiac arrhythmia, atrial fibrillation (AF) is managed across a wide spectrum of healthcare professionals, ranging broadly from general practitioners to emergency room practitioners, internal medicine specialists, and cardiologists. While many AF patients are asymptomatic and managed in the community, the risks of stroke and mortality are no different to symptomatic patients.^1^ The increasing mean age of the general population translates to greater healthcare costs for the UK National Health Service (NHS), with the greatest contribution from hospitalizations.^2^

Stroke prevention is central to the management of AF, but there remains a residual risk of adverse outcomes in AF patients despite oral anticoagulation. Indeed, mortality in anticoagulated AF patients remains high, but only 1 in 10 deaths are related to stroke, while 7 in 10 are cardiovascular related.^3^ Hence, current guidelines have proposed a more holistic or integrated approach to AF management.^4,5^ Such a streamlined approach is needed to ensure that the main pillars of AF care are delivered irrespective of how the patient is managed by different healthcare professionals.

Following confirmation of the diagnosis of AF, patients are characterized and evaluated using the 4S-AF scheme,^6^ i.e. stroke risk assessment (with the CHA_2_DS_2_VASc score), symptom severity (using the [European Heart Rhythm Association (EHRA) score], severity of burden (whether spontaneously terminating or permanent), and substrate (age, structural heart disease, and comorbidities). The patient is then treated according to the Atrial fibrillation Better Care (ABC) pathway,^7^ as follows: (A) avoid stroke with anticoagulation; (B) better symptom control with patient-centred symptom-directed decisions on rate or rhythm control; and (C) cardiovascular and comorbidity management, including lifestyle factors.

The ABC pathway is associated with improved clinical outcomes in numerous retrospective and prospective cohorts from different regions of the world,^8^ as well as post-hoc analysis from adjudicated outcomes from clinical trials.^9,10^ The mAFA-II clinical trial, which was a prospective cluster randomized trial, showed a significant reduction in the primary outcome with the ABC pathway intervention using an mHealth app, compared to usual care.^11,12^ Ongoing clinical trials are testing the impact of implementation of the ABC pathway in Europe (AFFIRMO^13^) and in rural China (MIRACLE-AF; NCT04622514).

In this study, our aim was to estimate the impact of implementing an ABC pathway in the UK between 2020 and 2040. We considered the impact in terms of morbidity (strokes and major bleeds), mortality, and associated healthcare costs from the health service perspective.

## Methods

This study used published evidence to estimate the annual prevalence of AF and the number of major non-fatal (strokes and major bleeds) and fatal events experienced by the population of AF patients. We also estimated healthcare costs associated with non-fatal events. We developed a mathematical model to estimate the expected effect of implementing an ABC pathway for AF patients. The approach to identifying parameters for the model is described below.

### PICO framework

The analysis conducted is summarized in the following Population, Intervention, Comparator, Outcomes (PICO) framework:

- Population: people with AF.
- Intervention: ABC pathway.
- Comparator: standard management (may include ‘A’, ‘B’, or ‘C’ but not all three).
- Outcomes: strokes, major bleeds, deaths (all-cause and cardiovascular), and healthcare costs associated with strokes and major bleeds.

### Prevalence of AF

We estimated the prevalence of AF in the UK population based on two routine data sources. The first was a publication of an analysis of a large primary care record database and reported the prevalence of AF in adults aged over 35 between 2000 and 2016 based on the records of over 5 million patients in the UK.^14^ The publication demonstrated that the trajectory of AF prevalence changed differently over time in different age groups. There was a relatively flat line in younger age groups and a much steeper slope for older age groups. This reflects both the accumulation of prevalent AF cases in older groups (as patients diagnosed with AF at a younger age become older) and a higher incidence rate of AF in older groups. Within each of three age groups (35–54, 55–74, and 75+ years), we extrapolated from the age-specific linear trends of the prevalence of AF between 2000 and 2016 to estimate the percentage prevalence within each age group for each year from 2020 to 2040.

The second data source was the official government figures for the UK population projection as compiled by the Office for National Statistics.^15^ We multiplied the expected percentage prevalence of AF for each year and age group by the projected population in the respective age group for the same year to estimate the number of patients with AF for each year up to 2040. This incorporates the changing demographics (i.e. growing proportion of older adults) of the UK population over time.

### Risks of fatal and non-fatal events without an ABC pathway

A systematic literature review and meta-analysis by Romiti *et al*.^8^ reported the number of strokes, major bleeds, deaths from all causes, and deaths from cardiovascular causes within study samples of AF patients. The follow-up periods varied between the studies included in the meta-analysis, so we calculated a one-year probability of each event for each study. We then used the weights attributed to each study as part of the meta-analysis to calculate a weighted mean one-year probability for each event type. The number of events was estimated for each calendar year by applying the event probability to the estimated number of people with AF in the respective year.

### Costs of non-fatal events

Our cost estimates relate to the health and social care perspective, as recommended by healthcare decision-makers in the UK.^16^ Our model included direct primary, secondary, and community healthcare costs associated with non-fatal events (strokes and major bleeds) beyond the index hospital admission. This is a key driver of costs associated with AF and stroke in particular. The source study used for the cost of stroke was from a large study of AF patients based in Oxford in England (The OXVASC (Oxford Vascular) Study).^17^ This study used a robust bottom-up methodology to capture and cost healthcare resource use associated with stroke in AF patients. In 2008/09 prices, they reported that 3-month costs associated with non-fatal strokes were £10 413 and annual costs in the post-acute period were £804 higher than before the stroke. We added 9 months of the annual post-acute cost difference to the 3-month acute cost to calculate a one-year cost associated with stroke (£11 016). The source study used for the cost of major bleeds used a UK large primary care dataset (Clinical Practice Research Datalink) linked with secondary care data (Hospital Episode Statistics) to estimate one-year costs associated with major gastrointestinal bleeds in patients with AF.^18^ They reported a mean cost of £3989 in 2017/18 prices. The healthcare costs associated with strokes and major bleeds were inflated from source papers to 2020/21 GBP (£) using the NHS ‘Pay and Prices’ and ‘Cost Inflation’ Indices.^19^ We multiplied these costs to the estimated number of events in 2020 to estimate associated healthcare costs. At the time of the analysis, inflation rates in the UK (and across much of the globe) were unusually high and unstable. The government's target for inflation remains at 2%.^20^ We assumed that it would take until 2030 to reach this level where it would remain until 2040. For the years between 2020 and 2030, we applied the following interest rate to the cost of the respective event from the previous year: 2021—3%; 2022 and 2023—10%; 2024–26—5%; and 2027–29—3%.

### Impact of implementing an ABC care pathway

We estimated the impact of implementing an ABC pathway on the number of fatal and non-fatal events and associated healthcare costs based on the findings reported in the Romiti *et al*.’s^8^ meta-analysis. They reported a pooled odds ratio (OR) for each event type among AF patients who received ABC care vs. AF patients who did not receive ABC care. We multiplied each OR by the respective event probability in the absence of ABC care to estimate the event probability for patients receiving ABC care. We subtracted the number (and cost) of events estimated for patients receiving ABC care from those not receiving ABC care as a proxy for the impact of ABC care. However, this assumes a 100% level of fidelity or implementation to ABC care and the meta-analysis reported that the prevalence of ABC implementation management across the included studies was only around 20%. Therefore, we also estimated the impact of ABC pathway management at 20%, 30%, 40%, 50%, 60%, 70%, 80%, and 90% implementation.

## Results

*Table 1* summarizes the model parameters used to estimate the population prevalence of AF up to 2040, the number of fatal and non-fatal events, associated costs, and impact of implementing the ABC pathway.

**Table 1:** Parameters used to estimate the number of patients with AF, number of fatal and non-fatal events, associated costs, and the impact of implementing ABC care pathway

Description	Parameter	Source
Prevalence of AF		
Population percentage prevalence of AF—annual data were used in the analysis; selected years are presented here (all years are shown in Supplementary material online)	2020 35–54: 0.35% 55–74: 3.27% 75+: 13.82% 2030 35–54: 0.42% 55–74: 3.76% 75+: 19.51% 2040 35–54: 0.49% 55–74: 4.26% 75+: 23.29%	Derived from Adderley *et al*.^14^
Projected UK population—annual data were used in the analysis; selected years are presented here (all years are shown in Supplementary material online)	Thousands of people 2025 35–54: 17 298 55–74: 15 637 75+: 6599 2030 35–54: 17 543 55–74: 16 380 75+: 7309 2035 35–54: 17 814 55–74: 16 435 75+: 7976 2040 35–54: 17 584 55–74: 16 326 75+: 8911	ONS^15^
Risk of fatal and non-fatal events (1-year probability)		
Strokes	0.014	Romiti *et al*.^8^ Additional calculations
Major bleeds	0.030	
Death from all causes	0.049	
Deaths from cardiovascular causes	0.023	
Unit costs		
Stroke: one-year costs including acute (0–3 months) and post-acute (4–12 months) healthcare in patients with AF	£13 508	Luengo-Fernandez *et al*.^31^ PSSRU unit costs^19^
Major bleed: one-year healthcare costs associated with gastrointestinal bleeds in patients with AF	£4297	Ramagopalan *et al*.^18^ PSSRU unit costs^19^
Impact of implementing the ABC pathway (odds ratio)		
Strokes	0.55(95% CI 0.37–0.82)	Romiti *et al*.^8^
Major bleeds	0.69(95% CI 0.51–0.94)	
Death from all causes	0.42(95% CI 0.31–0.56)	
Deaths from cardiovascular causes	0.37(95% CI 0.23–0.58)	
CI: confidence interval.		

**Figure 1:**
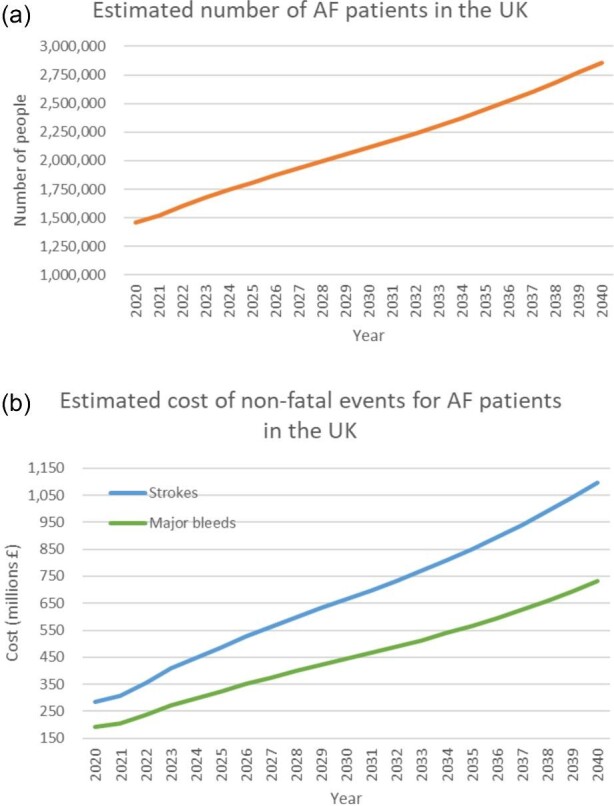
Estimated number of AF patients in the UK between 2020 and 2040.

*Figure 1* shows the estimated number of AF patients in the UK between 2020 and 2040 (panel a) and the costs associated with strokes and major bleeds in this population (panel b). The graph shows a clear increase that reflects the growing size and ageing of the population, increasing population percentage prevalence of AF, and increasing healthcare costs over time.

Supplementary material online, *Figure S1* shows trends over time in reported (2000–16) and estimated (2017–40) prevalence of AF in the UK using data published by Adderley *et al*.^14^ for 2000–16 with linear extrapolation within each age group for 2017–40.

In 2020, there were an estimated 1 463 538 AF patients, resulting in £286 million of stroke care and £191 million of care related to bleeds annually. By 2030, it is expected that there will be 2 115 332 AF patients, resulting in £666 million of stroke care and £444 million of care related to bleeds. By 2040, this is expected to rise to 2 856 489 AF patients, with £1 096 million of stroke care and £731 million of care related to bleeds for that year.

The estimated impact of ABC pathway management for each year from 2020 to 2040 is reported in full for each level of implementation in Supplementary material online, *Tables S1–S11*.

**Figure 2:**
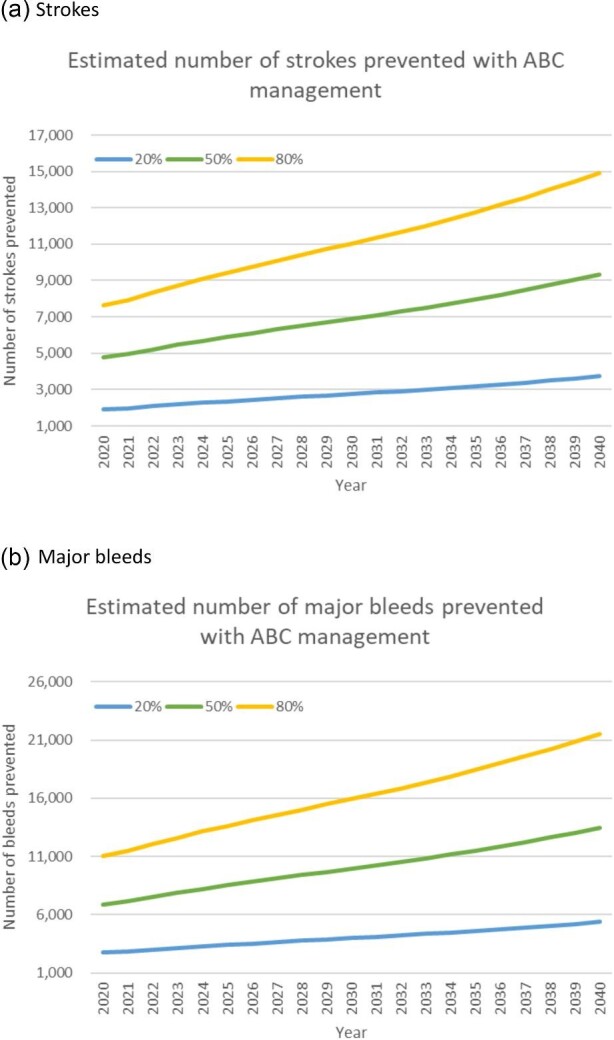
Estimated number of strokes and major bleeds prevented by ABC management of AF patients.

*Figure 2* shows the estimated number of strokes (panel a) and major bleeds (panel b) prevented for three different levels of implementation of ABC pathway-based care (20, 50, and 80% of AF patients managed according to the ABC pathway) between 2020 and 2040.

**Figure 3:**
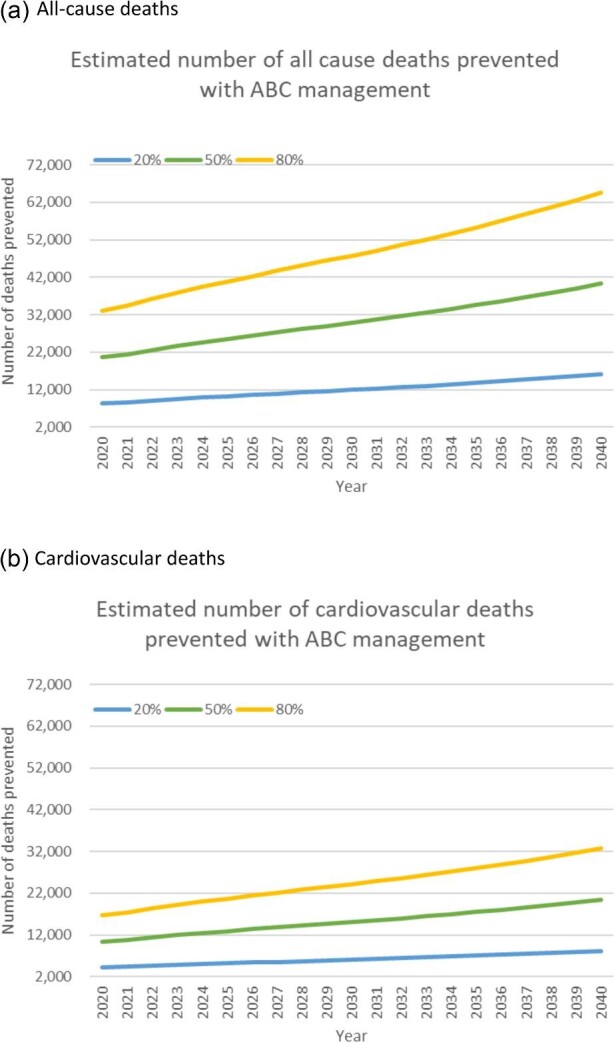
Estimated number of deaths bleeds prevented by ABC management of AF patients.

*Figure 3* shows the estimated number of all cause deaths (panel a) and cardiovascular deaths (panel b) prevented for three different levels of ABC care (20, 50, and 80% of AF patients managed according to the ABC pathway) between 2020 and 2040.

*Table 2* summarizes the estimated impact of ABC on each of the outcomes explored in 2030 and 2040. The differences between the two years reflect the increasing prevalence of AF and increasing healthcare costs. In 2030, between 2758 and 13 790 strokes and between 3983 and 19 913 major bleeds could be prevented. The associated reduction in healthcare costs of this is estimated to be between £87.4 and £437.1 million. In 2030, implementation of ABC pathway management could prevent between 11 945 and 59 726 deaths, around half of which would be due to cardiovascular causes. By 2040, the reduction in healthcare costs is estimated to be between £143.9 and £719.6 million and reduction in deaths is estimated to be between 16 131 and 80 653 for the year.

**Table 2:** Estimated impact of ABC management on outcomes and costs for AF patients in 2030 and 2040

	% of AF patients managed on ABC pathway								
	20	30	40	50	60	70	80	90	100
2030									
Strokes prevented	2758	4137	5516	6895	8274	9653	11 032	12 411	13 790
Cost reduction (£)—strokes	59 908 339	89 862 509	119 816 679	149 770 848	179 725 018	209 679 188	239 633 357	269 587 527	299 541 697
Bleeds prevented	3983	5974	7965	9956	11 948	13 939	15 930	17 921	19 913
Cost reduction (£)—bleeds	27 518 672	41 278 008	55 037 344	68 796 680	82 556 015	96 315 351	110 074 687	123 834 023	137 593 359
*Cost reduction (£)—total*	*87 427 011*	*131 140 517*	*174 854 022*	*218 567 528*	*262 281 033*	*305 994 539*	*349 708 044*	*393 421 550*	*437 135 056*
Deaths prevented (all cause)	11 945	17 918	23 890	29 863	35 836	41 808	47 781	53 754	59 726
Deaths prevented (CV)	6043	9064	12 086	15 107	18 129	21 150	24 172	27 193	30 214
2040									
Strokes prevented	3724	5586	7449	9311	11 173	13 035	14 897	16 759	18 622
Cost reduction (£)—strokes	98 615 026	147 922 539	197 230 052	246 537 565	295 845 078	345 152 591	394 460 104	443 767 617	493 075 130
Bleeds prevented	5378	8067	10 756	13 445	16 134	18 823	21 512	24 201	26 890
Cost reduction (£)—bleeds	45 298 444	67 947 666	90 596 888	113 246 109	135 895 331	158 544 553	181 193 775	203 842 997	226 492 219
*Cost reduction (£)—total*	*143 913 470*	*215 870 205*	*287 826 940*	*359 783 675*	*431 740 409*	*503 697 144*	*575 653 879*	*647 610 614*	*719 567 349*
Deaths prevented (all cause)	16 131	24 196	32 261	40 326	48 392	56 457	64 522	72 587	80 653
Deaths prevented (CV)	8160	12 240	16 320	20 400	24 481	28 561	32 641	36 721	40 801

CV = cardiovascular

## Discussion

In this analysis, we estimate that by 2040 there will be 2 856 489 AF patients, with £1096 million of stroke healthcare and £731 million of healthcare related to bleeds that year in the UK. In 2040, the ABC pathway could prevent between 3724 and 18 622 strokes and between 5378 and 26 890 bleeds, and save between 16 131 and 80 653 lives, depending on the proportion of patients managed on the pathway. This would equate to healthcare cost reductions of between £143.9 and £719.6 million.

As the population prevalence of AF increases over time, enhanced patient management based on the ABC pathway will become even more vitally important to improving patient outcomes and making efficient use of health service resources. Our prior modelling analysis estimated that given the number of newly diagnosed AF patients at age 65 will rise over the decade between 2020 and 2030, screening and treatment of AF has the potential to substantially reduce the health and social care costs of AF-related stroke in the NHS.^21^

There is limited existing evidence regarding the cost-effectiveness of the ABC pathway. A study from China used data from the mAFA-II trial to estimate the cost-effectiveness of using mHealth as a means of streamlining and integrating care for AF patients via the ABC pathway, from the perspective of a public healthcare provider in China.^22^ They reported that this was likely to be cost-effective over 30 years compared with usual care. Although not typical ABC pathway management, the SAFETY program in Australia reported that providing AF patients with a structured post-discharge package of coordinated care had a high probability of being cost-effective over a lifetime horizon.^23^

In the UK context, the components of ABC pathway-based care that are likely to result in additional use of NHS resources may well already be in place, as evident in the NHS Health Checks and CVD Prevention Strategy focused on ‘atrial fibrillation, blood pressure, and cholesterol’^24^ and efforts to prevent AF-related strokes (‘Detect, Protect, Perfect’).^25^ Also, it is worth reemphasizing that the core of the collaborative care element of the ABC pathway is the improved communication and collaboration between healthcare professionals, whether general practitioners or hospital doctors. This should really just be made part of optimized guideline-directed ‘usual care’ already, so in theory should not require additional resources. Indeed, guideline-adherent management has been associated with improved clinical outcomes when managing patients with AF.^26–28^

### Limitations

As is typical for this type of analysis, it was necessary to make assumptions to generate estimates from published data. A key assumption is that the longitudinal trend in the prevalence of AF would continue along the same trajectory as has been observed in routine primary care data between 2000 and 2016. We have also assumed that the prevalence of AF in the UK (England, Scotland, Wales, and Northern Ireland) is the same as for England.

Our cost estimates do not account for any increases in healthcare costs required to provide AF patients with ABC pathway management. We also do not account for increases in AF detection with screening, which may become more prevalent over the years. Nonetheless, systematic AF screening is not currently standard care in the UK, given current recommendations from the UK National Screening Programme^29^ and others.^30^ We anticipate that the impact of screening may be increased costs associated with managing more AF patients, but decreased costs in other areas as we identify more cases at earlier stages and are able to support patients to live well for longer with AF.

We have included one-year costs for strokes and bleeds as we have estimated annual snapshots of costs, but recognize that for stroke in particular there are likely to be longer-term increases in health and social care.^31^ If people move into residential care following a stroke, there is likely to be a substantial cost associated with this. This is relevant given that AF-related strokes are more likely to be disabling, with lower chance of discharge to the patient's own home.^32^ We have also not included costs from a broader societal perspective, such as those associated with informal care (i.e. unpaid care provided by family members or volunteers) or lost earnings.

We recognize that our analysis does not account for the direct cost of ABC pathway management per se. However, the present analysis is not an economic evaluation of ABC pathway management. Rather, our analysis provides a guide for commissioners and policymakers who are interested in implementing it as to the potential healthcare cost savings.

### Conclusion

We estimate that there will be a substantial healthcare burden of AF patients, from strokes, bleeds, and mortality in the UK NHS over the next decades. If patients are managed with a holistic or integrated care approach based on the ABC pathway, this management approach could prevent strokes and bleeds, and save lives equating to cost reductions of between £143.9 million and £719.6 million in 2040.

## Supplementary Material

qcad055_Supplemental_File

## Notes

**Conflict of interest: ** E.C.: none declared. G.Y.H.L. is a consultant and speaker for BMS/Pfizer, Boehringer Ingelheim, Daiichi Sankyo, and Anthos. No fees are received personally. G.Y.H.L. is a National Institute for Health and Care Research (NIHR) Senior Investigator and co-principal investigator of the AFFIRMO project on multimorbidity in atrial fibrillation, which has received funding from the European Union's Horizon 2020 research and innovation programme under grant agreement no. 899871.

## Data availability

All data relevant to the study are included in the article or uploaded as supplementary information.

## References

[1] Wallenhorst C, Martinez C, Freedman B. Risk of ischemic stroke in asymptomatic atrial fibrillation incidentally detected in primary care compared with other clinical presentations. Thromb Haemost 2022;122:277–285. 34192776 10.1055/a-1541-3885

[2] Burdett P, Lip GYH. Atrial fibrillation in the UK: predicting costs of an emerging epidemic recognizing and forecasting the cost drivers of atrial fibrillation-related costs. Eur Heart J Qual Care Clin Outcomes 2022;8:187–194. 33346822 10.1093/ehjqcco/qcaa093

[3] Pokorney SD, Piccini JP, Stevens SR, Patel MR, Pieper KS, Halperin JL et al. Cause of death and predictors of all-cause mortality in anticoagulated patients with nonvalvular atrial fibrillation: data From ROCKET AF. J Am Heart Assoc 2016;5:e002197. 26955859 10.1161/JAHA.115.002197 PMC4943233

[4] Chao TF, Joung B, Takahashi Y, Lim TW, Choi EK, Chan YH et al. 2021 Focused update consensus guidelines of the Asia Pacific Heart Rhythm Society on stroke prevention in atrial fibrillation: executive summary. Thromb Haemost 2022;122:20–47. 34773920 10.1055/s-0041-1739411 PMC8763451

[5] Hindricks G, Potpara T, Dagres N, Arbelo E, Bax JJ, Blomstrom-Lundqvist C et al. 2020 ESC Guidelines for the diagnosis and management of atrial fibrillation developed in collaboration with the European Association for Cardio-Thoracic Surgery (EACTS): the Task Force for the diagnosis and management of atrial fibrillation of the European Society of Cardiology (ESC) developed with the special contribution of the European Heart Rhythm Association (EHRA) of the ESC. Eur Heart J 2021;42:373–498. 32860505 10.1093/eurheartj/ehaa612

[6] Potpara TS, Lip GYH, Blomstrom-Lundqvist C, Boriani G, Van Gelder IC, Heidbuchel H et al. The 4S-AF scheme (stroke risk; symptoms; severity of burden; substrate): a novel approach to in-depth characterization (rather than classification) of atrial fibrillation. Thromb Haemost 2021;121:270–278. 32838473 10.1055/s-0040-1716408

[7] Lip GYH . The ABC pathway: an integrated approach to improve AF management. Nat Rev Cardiol 2017;14:627–628. 28960189 10.1038/nrcardio.2017.153

[8] Romiti GF, Pastori D, Rivera-Caravaca JM, Ding WY, Gue YX, Menichelli D et al. Adherence to the ‘Atrial fibrillation Better Care’ pathway in patients with atrial fibrillation: impact on clinical outcomes: a systematic review and meta-analysis of 285,000 patients. Thromb Haemost 2022;122:406–414. 34020488 10.1055/a-1515-9630

[9] Patel SM, Palazzolo MG, Murphy SA, Antman EM, Braunwald E, Lanz HJ et al. Evaluation of the atrial fibrillation better care pathway in the ENGAGE AF-TIMI 48 trial. Europace 2022;24:1730–1738. 36017608 10.1093/europace/euac082

[10] Proietti M, Romiti GF, Olshansky B, Lane DA, Lip GYH. Comprehensive management with the ABC (Atrial fibrillation Better Care) pathway in clinically complex patients with atrial fibrillation: a post hoc ancillary analysis from the AFFIRM trial. J Am Heart Assoc 2020;9:e014932. 32370588 10.1161/JAHA.119.014932 PMC7660878

[11] Guo Y, Lane DA, Wang L, Zhang H, Wang H, Zhang W et al. mAF-App II Trial Investigators. Mobile health technology to improve care for patients with atrial fibrillation. J Am Coll Cardiol 2020;75:1523–1534. 32241367 10.1016/j.jacc.2020.01.052

[12] Romiti GF, Guo Y, Corica B, Proietti M, Zhang H, Lip GYH, mAF-App II Trial Investigators. Mobile health-technology-integrated care for atrial fibrillation: a win ratio analysis from the mAFA-II randomized clinical trial. Thromb Haemost 2023. 10.1055/s-0043-1769612 37247623

[13] Johnsen SP, Proietti M, Maggioni AP, Lip GYH. A multinational European network to implement integrated care in elderly multimorbid atrial fibrillation patients: the AFFIRMO Consortium. Eur Heart J 2022;43:2916–2918. 35598035 10.1093/eurheartj/ehac265

[14] Adderley NJ, Ryan R, Nirantharakumar K, Marshall T. Prevalence and treatment of atrial fibrillation in UK general practice from 2000 to 2016. Heart 2019;105:27–33. 29991504 10.1136/heartjnl-2018-312977

[15] Principal projection—UK population in age groups .Accessed 12 August 2023. https://www.ons.gov.uk/peoplepopulationandcommunity/populationandmigration/populationprojections/datasets/tablea21principalprojectionukpopulationinagegroups.

[16] NICE . NICE. Health Technology Evaluations: The Manual. London: NICE; 2022.

[17] Luengo-Fernandez R, Yiin GS, Gray AM, Rothwell PM. Population-based study of acute- and long-term care costs after stroke in patients with AF. Int J Stroke 2013;8:308–314. 22568484 10.1111/j.1747-4949.2012.00812.x PMC6016735

[18] Ramagopalan SV, Samnaliev M, Weir S, Sammon CJ, Carroll R, Alikhan R. Costs of gastrointestinal bleeding events in atrial fibrillation: a UK Clinical Practice Research Datalink study. Future Cardiol 2019;15:367–375. 31347934 10.2217/fca-2019-0033

[19] Jones K, Burns A. Unit Costs of Health and Social Care 2021. Canterbury: Personal Social Services Research Unit, University of Kent; 2021. 10.22024/UniKent/01.02.92342.

[20] Bank of England Monetary policy . Accessed 12 August 2023. https://www.bankofengland.co.uk/monetary-policy.

[21] Burdett P, Lip GYH. Targeted vs. full population screening costs for incident atrial fibrillation and AF-related stroke for a healthy population aged 65 years in the United Kingdom. Eur Heart J Qual Care Clin Outcomes 2022;8:892–898. 35138372 10.1093/ehjqcco/qcac005 PMC9670327

[22] Luo X, Xu W, Ming WK, Jiang X, Yuan Q, Lai H et al. Cost-effectiveness of mobile health-based integrated care for atrial fibrillation: model development and data analysis. J Med Internet Res 2022;24:e29408. 35438646 10.2196/29408 PMC9066334

[23] Gao L, Scuffham P, Ball J, Stewart S, Byrnes J. Long-term cost-effectiveness of a disease management program for patients with atrial fibrillation compared to standard care—a multi-state survival model based on a randomized controlled trial. J Med Econ 2021;24:87–95. 33406944 10.1080/13696998.2020.1860371

[24] Size of the Prize and NHS Health Check factsheet . Accessed 12 August 2023. https://www.healthcheck.nhs.uk/commissioners-and-providers/data/size-of-the-prize-and-nhs-health-check-factsheet/.

[25] Atrial fibrillation (AF) toolkit: working together to prevent AF-related strokes . Accessed 12 August 2023. https://aftoolkit.co.uk

[26] Nieuwlaat R, Olsson SB, Lip GY, Camm AJ, Breithardt G, Capucci A et al. Guideline-adherent antithrombotic treatment is associated with improved outcomes compared with undertreatment in high-risk patients with atrial fibrillation. The Euro Heart Survey on Atrial Fibrillation. Am Heart J 2007;153:1006–1012. 17540203 10.1016/j.ahj.2007.03.008

[27] Lip GY, Laroche C, Popescu MI, Rasmussen LH, Vitali-Serdoz L, Dan GA et al. Improved outcomes with European Society of Cardiology guideline-adherent antithrombotic treatment in high-risk patients with atrial fibrillation: a report from the EORP-AF General Pilot Registry. Europace 2015;17:1777–1786. 26321406 10.1093/europace/euv269

[28] Gorin L, Fauchier L, Nonin E, Charbonnier B, Babuty D, Lip GYH. Prognosis and guideline-adherent antithrombotic treatment in patients with atrial fibrillation and atrial flutter: implications of undertreatment and overtreatment in real-life clinical practice; the Loire Valley Atrial Fibrillation Project. Chest 2011;140:911–917. 21436246 10.1378/chest.10-2436

[29] King S, Fitzgerald S, Bartlett C. Evidence summary for screening for atrial fibrillation in adults: external review against programme appraisal criteria for the UK national screening Committee: UK national screening Committee. 2019. Accessed 12 August 2023. https://view-health-screening-recommendations.service.gov.uk/atrial-fibrillation/.

[30] Force USPST, Davidson KW, Barry MJ, Mangione CM, Cabana M, Caughey AB et al. Screening for atrial fibrillation: US Preventive Services Task Force recommendation statement. J Am Med Assoc 2022;327:360–367. 10.1001/jama.2021.23732 35076659

[31] Luengo-Fernandez R, Violato M, Candio P, Leal J. Economic burden of stroke across Europe: a population-based cost analysis. Eur Stroke J 2020;5:17–25. 32232166 10.1177/2396987319883160 PMC7092742

[32] Lip G, Freedman B, De Caterina R, Potpara TS. Stroke prevention in atrial fibrillation: past, present and future. Comparing the guidelines and practical decision-making. Thromb Haemost 2017;117:1230–1239. 28597905 10.1160/TH16-11-0876

