# Supplementary material for: Estimating the impact of implementing an integrated care management approach with Atrial fibrillation Better Care (ABC) pathway for patients with atrial fibrillation in England from 2020 to 2040
Source: Eur Heart J Qual Care Clin Outcomes. 2023 Sep 11;10(4):326–33. doi: 10.1093/ehjqcco/qcad055 (PMC11187718; doi:10.1093/ehjqcco/qcad055)
Supplement: qcad055_Supplemental_File [file qcad055_supplemental_file.docx]

**SUPPLEMTARY MATERIAL**

**Table S1.** Predicted UK population by age group – summary of Office for National Statistics (ONS) data

| **Age group** | **2020** | **2021** | **2022** | **2023** | **2024** | **2025** | **2026** | **2027** | **2028** | **2029** | **2030** |
| --- | --- | --- | --- | --- | --- | --- | --- | --- | --- | --- | --- |
| **35-54** | 17,416 | 17,373 | 17,325 | 17,314 | 17,298 | 17,313 | 17,327 | 17,355 | 17,396 | 17,473 | 17,543 |
| **55-74** | 15,086 | 15,305 | 15,365 | 15,482 | 15,637 | 15,788 | 15,974 | 16,138 | 16,250 | 16,320 | 16,380 |
| **75+** | 5,789 | 5,920 | 6,202 | 6,424 | 6,599 | 6,747 | 6,871 | 6,980 | 7,095 | 7,208 | 7,309 |
|  |  |  |  |  |  |  |  |  |  |  |  |
|  | **2031** | **2032** | **2033** | **2034** | **2035** | **2036** | **2037** | **2038** | **2039** | **2040** |  |
| **35-54** | 17,634 | 17,746 | 17,827 | 17,847 | 17,814 | 17,766 | 17,701 | 17,646 | 17,619 | 17,584 |  |
| **55-74** | 16,404 | 16,393 | 16,372 | 16,393 | 16,435 | 16,449 | 16,435 | 16,406 | 16,358 | 16,326 |  |
| **75+** | 7,422 | 7,547 | 7,686 | 7,828 | 7,976 | 8,142 | 8,325 | 8,515 | 8,713 | 8,911 |  |

**Table S2.** Estimated percentage prevalence of AF between 2020 and 2040 – derived from Adderley et al 2019.

| **Age group** | **2020** | **2021** | **2022** | **2023** | **2024** | **2025** | **2026** | **2027** | **2028** | **2029** | **2030** |
| --- | --- | --- | --- | --- | --- | --- | --- | --- | --- | --- | --- |
| **35-54** | 0.35% | 0.35% | 0.36% | 0.37% | 0.37% | 0.38% | 0.39% | 0.40% | 0.40% | 0.41% | 0.42% |
| **55-74** | 3.27% | 3.32% | 3.37% | 3.42% | 3.46% | 3.51% | 3.56% | 3.61% | 3.66% | 3.71% | 3.76% |
| **75+** | 15.73% | 16.11% | 16.48% | 16.86% | 17.24% | 17.62% | 18.00% | 18.38% | 18.75% | 19.13% | 19.51% |
|  |  |  |  |  |  |  |  |  |  |  |  |
|  | **2031** | **2032** | **2033** | **2034** | **2035** | **2036** | **2037** | **2038** | **2039** | **2040** |  |
| **35-54** | 0.42% | 0.43% | 0.44% | 0.45% | 0.45% | 0.46% | 0.47% | 0.47% | 0.48% | 0.49% |  |
| **55-74** | 3.81% | 3.86% | 3.91% | 3.96% | 4.01% | 4.06% | 4.11% | 4.16% | 4.21% | 4.26% |  |
| **75+** | 19.89% | 20.27% | 20.65% | 21.02% | 21.40% | 21.78% | 22.16% | 22.54% | 22.92% | 23.29% |  |

**Figure S1.** Trends over time in reported (2000-2016) and estimated (2017-2040) prevalence of AF in the UK

| 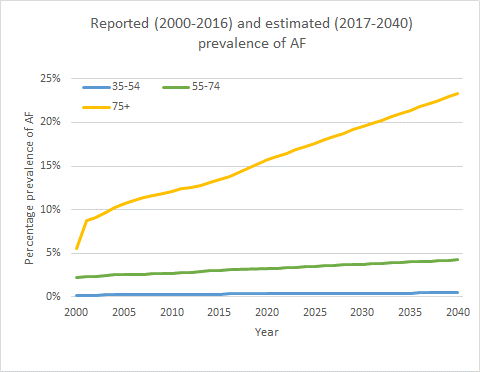  Data published in Adderley et al 2019 for 2000-2016 with linear extrapolation within each age group for 2017-2040 |
| --- |

**Table S3.**

Estimated Impact of ABC management on outcomes and costs for AF patients between 2020 and 2040 – 20% of patients on ABC pathway

|  | **2020** | **2021** | **2022** | **2023** | **2024** | **2025** | **2026** | **2027** | **2028** | **2029** | **2030** |
| --- | --- | --- | --- | --- | --- | --- | --- | --- | --- | --- | --- |
| **AF patients** | 1,463,538 | 1,522,388 | 1,601,810 | 1,675,568 | 1,744,290 | 1,809,579 | 1,873,197 | 1,934,384 | 1,995,919 | 2,056,528 | 2,115,332 |
| **Strokes prevented** | 1,908 | 1,985 | 2,088 | 2,185 | 2,274 | 2,359 | 2,442 | 2,522 | 2,602 | 2,681 | 2,758 |
| **Bleeds prevented** | 2,755 | 2,866 | 3,016 | 3,155 | 3,284 | 3,407 | 3,527 | 3,642 | 3,758 | 3,872 | 3,983 |
| **Cost reduction(£)** | 37,615,665 | 40,302,056 | 46,645,058 | 53,672,204 | 58,667,197 | 63,906,290 | 69,460,609 | 73,881,396 | 78,518,604 | 83,330,021 | 87,427,011 |
| **Deaths prevented (all cause)** | 8,265 | 8,597 | 9,045 | 9,462 | 9,850 | 10,219 | 10,578 | 10,923 | 11,271 | 11,613 | 11,945 |
| **Deaths prevented(CV)** | 4,181 | 4,349 | 4,576 | 4,787 | 4,983 | 5,169 | 5,351 | 5,526 | 5,702 | 5,875 | 6,043 |
|  |  |  |  |  |  |  |  |  |  |  |  |
|  | **2031** | **2032** | **2033** | **2034** | **2035** | **2036** | **2037** | **2038** | **2039** | **2040** |  |
| **AF patients** | 2,176,114 | 2,239,015 | 2,305,142 | 2,374,498 | 2,446,592 | 2,522,857 | 2,602,541 | 2,684,847 | 2,769,683 | 2,856,489 |  |
| **Strokes prevented** | 2,837 | 2,919 | 3,005 | 3,096 | 3,190 | 3,289 | 3,393 | 3,501 | 3,611 | 3,724 |  |
| **Bleeds prevented** | 4,097 | 4,215 | 4,340 | 4,470 | 4,606 | 4,750 | 4,900 | 5,055 | 5,214 | 5,378 |  |
| **Cost reduction (£)** | 91,737,945 | 96,277,415 | 101,103,296 | 106,228,141 | 111,642,488 | 117,425,031 | 123,556,564 | 130,013,370 | 136,803,996 | 143,913,470 |  |
| **Deaths prevented (all cause)** | 12,288 | 12,644 | 13,017 | 13,409 | 13,816 | 14,247 | 14,696 | 15,161 | 15,640 | 16,131 |  |
| **Deaths prevented(CV)** | 6,217 | 6,396 | 6,585 | 6,783 | 6,989 | 7,207 | 7,435 | 7,670 | 7,912 | 8,160 |  |

**Table S4.**

Estimated Impact of ABC management on outcomes and costs for AF patients between 2020 and 2040 – 30% of patients on ABC pathway

|  | **2020** | **2021** | **2022** | **2023** | **2024** | **2025** | **2026** | **2027** | **2028** | **2029** | **2030** |
| --- | --- | --- | --- | --- | --- | --- | --- | --- | --- | --- | --- |
| **Strokes prevented** | 2,862 | 2,977 | 3,133 | 3,277 | 3,411 | 3,539 | 3,663 | 3,783 | 3,903 | 4,022 | 4,137 |
| **Bleeds prevented** | 4,133 | 4,299 | 4,524 | 4,732 | 4,926 | 5,110 | 5,290 | 5,463 | 5,637 | 5,808 | 5,974 |
| **Cost reduction (£)** | 56,423,498 | 60,453,084 | 69,967,587 | 80,508,306 | 88,000,795 | 95,859,435 | 104,190,914 | 110,822,093 | 117,777,905 | 124,995,032 | 131,140,517 |
| **Deaths prevented (all cause)** | 12,397 | 12,895 | 13,568 | 14,193 | 14,775 | 15,328 | 15,867 | 16,385 | 16,906 | 17,420 | 17,918 |
| **Deaths prevented (CV)** | 6,271 | 6,524 | 6,864 | 7,180 | 7,474 | 7,754 | 8,027 | 8,289 | 8,553 | 8,812 | 9,064 |
|  |  |  |  |  |  |  |  |  |  |  |  |
|  | **2031** | **2032** | **2033** | **2034** | **2035** | **2036** | **2037** | **2038** | **2039** | **2040** |  |
| **Strokes prevented** | 4,256 | 4,379 | 4,508 | 4,644 | 4,785 | 4,934 | 5,090 | 5,251 | 5,417 | 5,586 |  |
| **Bleeds prevented** | 6,145 | 6,323 | 6,510 | 6,706 | 6,909 | 7,125 | 7,350 | 7,582 | 7,822 | 8,067 |  |
| **Cost reduction (£)** | 137,606,918 | 144,416,122 | 151,654,944 | 159,342,211 | 167,463,732 | 176,137,547 | 185,334,846 | 195,020,055 | 205,205,994 | 215,870,205 |  |
| **Deaths prevented (all cause)** | 18,433 | 18,966 | 19,526 | 20,113 | 20,724 | 21,370 | 22,045 | 22,742 | 23,461 | 24,196 |  |
| **Deaths prevented (CV)** | 9,325 | 9,594 | 9,878 | 10,175 | 10,484 | 10,811 | 11,152 | 11,505 | 11,868 | 12,240 |  |

**Table S5.**

Estimated Impact of ABC management on outcomes and costs for AF patients between 2020 and 2040 – 40% of patients on ABC pathway

|  | **2020** | **2021** | **2022** | **2023** | **2024** | **2025** | **2026** | **2027** | **2028** | **2029** | **2030** |
| --- | --- | --- | --- | --- | --- | --- | --- | --- | --- | --- | --- |
| **Strokes prevented** | 3,816 | 3,970 | 4,177 | 4,369 | 4,548 | 4,719 | 4,885 | 5,044 | 5,205 | 5,363 | 5,516 |
| **Bleeds prevented** | 5,511 | 5,732 | 6,031 | 6,309 | 6,568 | 6,814 | 7,053 | 7,284 | 7,515 | 7,744 | 7,965 |
| **Cost reduction (£)** | 75,231,330 | 80,604,112 | 93,290,116 | 107,344,407 | 117,334,394 | 127,812,580 | 138,921,219 | 147,762,791 | 157,037,207 | 166,660,043 | 174,854,022 |
| **Deaths prevented (all cause)** | 16,529 | 17,194 | 18,091 | 18,924 | 19,700 | 20,437 | 21,156 | 21,847 | 22,542 | 23,226 | 23,890 |
| **Deaths prevented (CV)** | 8,362 | 8,698 | 9,152 | 9,573 | 9,966 | 10,339 | 10,702 | 11,052 | 11,404 | 11,750 | 12,086 |
|  |  |  |  |  |  |  |  |  |  |  |  |
|  | **2031** | **2032** | **2033** | **2034** | **2035** | **2036** | **2037** | **2038** | **2039** | **2040** |  |
| **Strokes prevented** | 5,674 | 5,839 | 6,011 | 6,192 | 6,380 | 6,579 | 6,786 | 7,001 | 7,222 | 7,449 |  |
| **Bleeds prevented** | 8,194 | 8,431 | 8,680 | 8,941 | 9,212 | 9,500 | 9,800 | 10,110 | 10,429 | 10,756 |  |
| **Cost reduction (£)** | 183,475,891 | 192,554,830 | 202,206,592 | 212,456,282 | 223,284,976 | 234,850,063 | 247,113,128 | 260,026,740 | 273,607,991 | 287,826,940 |  |
| **Deaths prevented (all cause)** | 24,577 | 25,287 | 26,034 | 26,817 | 27,632 | 28,493 | 29,393 | 30,323 | 31,281 | 32,261 |  |
| **Deaths prevented (CV)** | 12,433 | 12,792 | 13,170 | 13,567 | 13,978 | 14,414 | 14,869 | 15,340 | 15,824 | 16,320 |  |

**Table S6.**

Estimated Impact of ABC management on outcomes and costs for AF patients between 2020 and 2040 – 50% of patients on ABC pathway

|  | **2020** | **2021** | **2022** | **2023** | **2024** | **2025** | **2026** | **2027** | **2028** | **2029** | **2030** |
| --- | --- | --- | --- | --- | --- | --- | --- | --- | --- | --- | --- |
| **Strokes prevented** | 4,770 | 4,962 | 5,221 | 5,462 | 5,686 | 5,898 | 6,106 | 6,305 | 6,506 | 6,703 | 6,895 |
| **Bleeds prevented** | 6,889 | 7,165 | 7,539 | 7,886 | 8,210 | 8,517 | 8,817 | 9,105 | 9,394 | 9,680 | 9,956 |
| **Cost reduction (£)** | 94,039,163 | 100,755,140 | 116,612,645 | 134,180,509 | 146,667,992 | 159,765,724 | 173,651,524 | 184,703,489 | 196,296,509 | 208,325,054 | 218,567,528 |
| **Deaths prevented (all cause)** | 20,661 | 21,492 | 22,613 | 23,655 | 24,625 | 25,547 | 26,445 | 27,309 | 28,177 | 29,033 | 29,863 |
| **Deaths prevented (CV)** | 10,452 | 10,873 | 11,440 | 11,967 | 12,457 | 12,924 | 13,378 | 13,815 | 14,254 | 14,687 | 15,107 |
|  |  |  |  |  |  |  |  |  |  |  |  |
|  | **2031** | **2032** | **2033** | **2034** | **2035** | **2036** | **2037** | **2038** | **2039** | **2040** |  |
| **Strokes prevented** | 7,093 | 7,298 | 7,514 | 7,740 | 7,975 | 8,223 | 8,483 | 8,751 | 9,028 | 9,311 |  |
| **Bleeds prevented** | 10,242 | 10,538 | 10,850 | 11,176 | 11,516 | 11,874 | 12,250 | 12,637 | 13,036 | 13,445 |  |
| **Cost reduction (£)** | 229,344,864 | 240,693,537 | 252,758,240 | 265,570,352 | 279,106,220 | 293,562,579 | 308,891,410 | 325,033,425 | 342,009,989 | 359,783,675 |  |
| **Deaths prevented (all cause)** | 30,721 | 31,609 | 32,543 | 33,522 | 34,540 | 35,616 | 36,741 | 37,903 | 39,101 | 40,326 |  |
| **Deaths prevented (CV)** | 15,541 | 15,991 | 16,463 | 16,958 | 17,473 | 18,018 | 18,587 | 19,175 | 19,780 | 20,400 |  |

**Table S7**.

Estimated Impact of ABC management on outcomes and costs for AF patients between 2020 and 2040 – 60% of patients on ABC pathway

|  | **2020** | **2021** | **2022** | **2023** | **2024** | **2025** | **2026** | **2027** | **2028** | **2029** | **2030** |
| --- | --- | --- | --- | --- | --- | --- | --- | --- | --- | --- | --- |
| **Strokes prevented** | 5,725 | 5,955 | 6,265 | 6,554 | 6,823 | 7,078 | 7,327 | 7,566 | 7,807 | 8,044 | 8,274 |
| **Bleeds prevented** | 8,266 | 8,599 | 9,047 | 9,464 | 9,852 | 10,221 | 10,580 | 10,926 | 11,273 | 11,615 | 11,948 |
| **Cost reduction (£)** | 112,846,996 | 120,906,167 | 139,935,173 | 161,016,611 | 176,001,590 | 191,718,869 | 208,381,828 | 221,644,187 | 235,555,811 | 249,990,064 | 262,281,033 |
| **Deaths prevented (all cause)** | 24,794 | 25,791 | 27,136 | 28,386 | 29,550 | 30,656 | 31,734 | 32,770 | 33,813 | 34,840 | 35,836 |
| **Deaths prevented (CV)** | 12,543 | 13,047 | 13,728 | 14,360 | 14,949 | 15,508 | 16,054 | 16,578 | 17,105 | 17,625 | 18,129 |
|  |  |  |  |  |  |  |  |  |  |  |  |
|  | **2031** | **2032** | **2033** | **2034** | **2035** | **2036** | **2037** | **2038** | **2039** | **2040** |  |
| **Strokes prevented** | 8,512 | 8,758 | 9,016 | 9,288 | 9,570 | 9,868 | 10,180 | 10,502 | 10,833 | 11,173 |  |
| **Bleeds prevented** | 12,291 | 12,646 | 13,020 | 13,411 | 13,819 | 14,249 | 14,699 | 15,164 | 15,643 | 16,134 |  |
| **Cost reduction (£)** | 275,213,836 | 288,832,245 | 303,309,888 | 318,684,422 | 334,927,464 | 352,275,094 | 370,669,692 | 390,040,110 | 410,411,987 | 431,740,409 |  |
| **Deaths prevented (all cause)** | 36,865 | 37,931 | 39,051 | 40,226 | 41,448 | 42,740 | 44,089 | 45,484 | 46,921 | 48,392 |  |
| **Deaths prevented (CV)** | 18,650 | 19,189 | 19,755 | 20,350 | 20,968 | 21,621 | 22,304 | 23,010 | 23,737 | 24,481 |  |

**Table S8.**

Estimated Impact of ABC management on outcomes and costs for AF patients between 2020 and 2040 – 70% of patients on ABC pathway

|  | **2020** | **2021** | **2022** | **2023** | **2024** | **2025** | **2026** | **2027** | **2028** | **2029** | **2030** |
| --- | --- | --- | --- | --- | --- | --- | --- | --- | --- | --- | --- |
| **Strokes prevented** | 6,679 | 6,947 | 7,310 | 7,646 | 7,960 | 8,258 | 8,548 | 8,827 | 9,108 | 9,385 | 9,653 |
| **Bleeds prevented** | 9,644 | 10,032 | 10,555 | 11,041 | 11,494 | 11,924 | 12,343 | 12,747 | 13,152 | 13,551 | 13,939 |
| **Cost reduction (£)** | 131,654,828 | 141,057,195 | 163,257,702 | 187,852,713 | 205,335,189 | 223,672,014 | 243,112,133 | 258,584,885 | 274,815,113 | 291,655,075 | 305,994,539 |
| **Deaths prevented (all cause)** | 28,926 | 30,089 | 31,659 | 33,117 | 34,475 | 35,765 | 37,023 | 38,232 | 39,448 | 40,646 | 41,808 |
| **Deaths prevented (CV)** | 14,633 | 15,222 | 16,016 | 16,753 | 17,440 | 18,093 | 18,729 | 19,341 | 19,956 | 20,562 | 21,150 |
|  |  |  |  |  |  |  |  |  |  |  |  |
|  | **2031** | **2032** | **2033** | **2034** | **2035** | **2036** | **2037** | **2038** | **2039** | **2040** |  |
| **Strokes prevented** | 9,930 | 10,217 | 10,519 | 10,836 | 11,165 | 11,513 | 11,876 | 12,252 | 12,639 | 13,035 |  |
| **Bleeds prevented** | 14,339 | 14,754 | 15,190 | 15,647 | 16,122 | 16,624 | 17,149 | 17,692 | 18,251 | 18,823 |  |
| **Cost reduction (£)** | 321,082,809 | 336,970,952 | 353,861,536 | 371,798,493 | 390,748,708 | 410,987,610 | 432,447,974 | 455,046,795 | 478,813,985 | 503,697,144 |  |
| **Deaths prevented (all cause)** | 43,010 | 44,253 | 45,560 | 46,931 | 48,356 | 49,863 | 51,438 | 53,064 | 54,741 | 56,457 |  |
| **Deaths prevented (CV)** | 21,758 | 22,387 | 23,048 | 23,741 | 24,462 | 25,225 | 26,021 | 26,844 | 27,693 | 28,561 |  |

**Table S9.**

Estimated Impact of ABC management on outcomes and costs for AF patients between 2020 and 2040 – 80% of patients on ABC pathway

|  | **2020** | **2021** | **2022** | **2023** | **2024** | **2025** | **2026** | **2027** | **2028** | **2029** | **2030** |
| --- | --- | --- | --- | --- | --- | --- | --- | --- | --- | --- | --- |
| **Strokes prevented** | 7,633 | 7,940 | 8,354 | 8,739 | 9,097 | 9,437 | 9,769 | 10,088 | 10,409 | 10,725 | 11,032 |
| **Bleeds prevented** | 11,022 | 11,465 | 12,063 | 12,618 | 13,136 | 13,628 | 14,107 | 14,567 | 15,031 | 15,487 | 15,930 |
| **Cost reduction (£)** | 150,462,661 | 161,208,223 | 186,580,231 | 214,688,815 | 234,668,787 | 255,625,159 | 277,842,438 | 295,525,583 | 314,074,414 | 333,320,086 | 349,708,044 |
| **Deaths prevented (all cause)** | 33,058 | 34,388 | 36,182 | 37,848 | 39,400 | 40,875 | 42,312 | 43,694 | 45,084 | 46,453 | 47,781 |
| **Deaths prevented (CV)** | 16,724 | 17,396 | 18,304 | 19,146 | 19,932 | 20,678 | 21,405 | 22,104 | 22,807 | 23,500 | 24,172 |
|  |  |  |  |  |  |  |  |  |  |  |  |
|  | **2031** | **2032** | **2033** | **2034** | **2035** | **2036** | **2037** | **2038** | **2039** | **2040** |  |
| **Strokes prevented** | 11,349 | 11,677 | 12,022 | 12,384 | 12,760 | 13,157 | 13,573 | 14,002 | 14,445 | 14,897 |  |
| **Bleeds prevented** | 16,388 | 16,862 | 17,360 | 17,882 | 18,425 | 18,999 | 19,599 | 20,219 | 20,858 | 21,512 |  |
| **Cost reduction (£)** | 366,951,782 | 385,109,659 | 404,413,184 | 424,912,563 | 446,569,952 | 469,700,126 | 494,226,256 | 520,053,480 | 547,215,983 | 575,653,879 |  |
| **Deaths prevented (all cause)** | 49,154 | 50,575 | 52,068 | 53,635 | 55,263 | 56,986 | 58,786 | 60,645 | 62,561 | 64,522 |  |
| **Deaths prevented (CV)** | 24,866 | 25,585 | 26,341 | 27,133 | 27,957 | 28,828 | 29,739 | 30,679 | 31,649 | 32,641 |  |

**Table S10.**

Estimated Impact of ABC management on outcomes and costs for AF patients between 2020 and 2040 – 90% of patients on ABC pathway

|  | **2020** | **2021** | **2022** | **2023** | **2024** | **2025** | **2026** | **2027** | **2028** | **2029** | **2030** |
| --- | --- | --- | --- | --- | --- | --- | --- | --- | --- | --- | --- |
| **Strokes prevented** | 8,587 | 8,932 | 9,398 | 9,831 | 10,234 | 10,617 | 10,990 | 11,349 | 11,710 | 12,066 | 12,411 |
| **Bleeds prevented** | 12,399 | 12,898 | 13,571 | 14,196 | 14,778 | 15,331 | 15,870 | 16,388 | 16,910 | 17,423 | 17,921 |
| **Cost reduction (£)** | 169,270,493 | 181,359,251 | 209,902,760 | 241,524,917 | 264,002,386 | 287,578,304 | 312,572,743 | 332,466,280 | 353,333,716 | 374,985,097 | 393,421,550 |
| **Deaths prevented (all cause)** | 37,191 | 38,686 | 40,704 | 42,579 | 44,325 | 45,984 | 47,601 | 49,155 | 50,719 | 52,259 | 53,754 |
| **Deaths prevented (CV)** | 18,814 | 19,571 | 20,592 | 21,540 | 22,423 | 23,263 | 24,080 | 24,867 | 25,658 | 26,437 | 27,193 |
|  |  |  |  |  |  |  |  |  |  |  |  |
|  | **2031** | **2032** | **2033** | **2034** | **2035** | **2036** | **2037** | **2038** | **2039** | **2040** |  |
| **Strokes prevented** | 12,768 | 13,137 | 13,525 | 13,932 | 14,355 | 14,802 | 15,270 | 15,752 | 16,250 | 16,759 |  |
| **Bleeds prevented** | 18,436 | 18,969 | 19,530 | 20,117 | 20,728 | 21,374 | 22,049 | 22,746 | 23,465 | 24,201 |  |
| **Cost reduction (£)** | 412,820,755 | 433,248,367 | 454,964,832 | 478,026,633 | 502,391,196 | 528,412,642 | 556,004,538 | 585,060,165 | 615,617,981 | 647,610,614 |  |
| **Deaths prevented (all cause)** | 55,298 | 56,897 | 58,577 | 60,339 | 62,171 | 64,109 | 66,134 | 68,226 | 70,382 | 72,587 |  |
| **Deaths prevented (CV)** | 27,974 | 28,783 | 29,633 | 30,525 | 31,451 | 32,432 | 33,456 | 34,514 | 35,605 | 36,721 |  |

**Table S11.**

Estimated Impact of ABC management on outcomes and costs for AF patients between 2020 and 2040 – 100% of patients on ABC pathway

|  | **2020** | **2021** | **2022** | **2023** | **2024** | **2025** | **2026** | **2027** | **2028** | **2029** | **2030** |
| --- | --- | --- | --- | --- | --- | --- | --- | --- | --- | --- | --- |
| **Strokes prevented** | 9,541 | 9,925 | 10,442 | 10,923 | 11,371 | 11,797 | 12,211 | 12,610 | 13,012 | 13,407 | 13,790 |
| **Bleeds prevented** | 13,777 | 14,331 | 15,079 | 15,773 | 16,420 | 17,034 | 17,633 | 18,209 | 18,789 | 19,359 | 19,913 |
| **Cost reduction (£)** | 188,078,326 | 201,510,279 | 233,225,289 | 268,361,019 | 293,335,984 | 319,531,449 | 347,303,047 | 369,406,978 | 392,593,018 | 416,650,107 | 437,135,056 |
| **Deaths prevented (all cause)** | 41,323 | 42,984 | 45,227 | 47,310 | 49,250 | 51,093 | 52,890 | 54,617 | 56,355 | 58,066 | 59,726 |
| **Deaths prevented (CV)** | 20,905 | 21,745 | 22,880 | 23,933 | 24,915 | 25,847 | 26,756 | 27,630 | 28,509 | 29,375 | 30,214 |
|  |  |  |  |  |  |  |  |  |  |  |  |
|  | **2031** | **2032** | **2033** | **2034** | **2035** | **2036** | **2037** | **2038** | **2039** | **2040** |  |
| **Strokes prevented** | 14,186 | 14,596 | 15,027 | 15,480 | 15,949 | 16,447 | 16,966 | 17,503 | 18,056 | 18,622 |  |
| **Bleeds prevented** | 20,485 | 21,077 | 21,699 | 22,352 | 23,031 | 23,749 | 24,499 | 25,274 | 26,072 | 26,890 |  |
| **Cost reduction (£)** | 458,689,727 | 481,387,074 | 505,516,480 | 531,140,704 | 558,212,440 | 587,125,157 | 617,782,820 | 650,066,850 | 684,019,978 | 719,567,349 |  |
| **Deaths prevented (all cause)** | 61,442 | 63,218 | 65,085 | 67,044 | 69,079 | 71,233 | 73,482 | 75,806 | 78,202 | 80,653 |  |
| **Deaths prevented (CV)** | 31,083 | 31,981 | 32,926 | 33,916 | 34,946 | 36,035 | 37,174 | 38,349 | 39,561 | 40,801 |  |
